# Elucidating the Role of Serum tRF-31-U5YKFN8DYDZDD as a Novel Diagnostic Biomarker in Gastric Cancer (GC)

**DOI:** 10.3389/fonc.2021.723753

**Published:** 2021-08-23

**Authors:** Yuejiao Huang, Haiyan Zhang, Xinliang Gu, Shiyi Qin, Ming Zheng, Xiangrong Shi, Chunlei Peng, Shaoqing Ju

**Affiliations:** ^1^Department of Medical Oncology, Affiliated Hospital of Nantong University, Nantong, China; ^2^Medical School of Nantong University, Nantong, China; ^3^Department of Pathology, Affiliated Nantong Third Hospital of Nantong University, Nantong, China; ^4^Department of Laboratory Medicine, Affiliated Hospital of Nantong University, Nantong, China; ^5^Department of Medical Oncology, Affiliated Tumor Hospital of Nantong University, Nantong, China

**Keywords:** tRNA-derived small RNAs, tRF-31-U5YKFN8DYDZDD, diagnosis biomarker, gastric cancer, prognosis

## Abstract

**Background:**

Gastric cancer (GC) is one of the malignant tumors with the highest morbidity and mortality in the world. Early diagnosis combined with surgical treatment can significantly improve the prognosis of patients. Therefore, it is urgent to seek higher sensitivity and specificity biomarkers in GC. tRNA-derived small RNAs are a new non-coding small RNA that widely exists in tumor cells and body fluids. In this study, we explore the expression and biological significance of tRNA-derived small RNAs in GC.

**Materials and Methods:**

First of all, we screened the differentially expressed tRNA-derived small RNAs in tumor tissues by high-throughput sequencing. Agarose gel electrophoresis (AGE), Sanger sequencing, and Nuclear and Cytoplasmic RNA Separation Assay were used to screen tRF-31-U5YKFN8DYDZDD as a potential tumor biomarker for the diagnosis of GC. Then, we detected the different expressions of tRF-31-U5YKFN8DYDZDD in 24 pairs of GC and paracancerous tissues, the serum of 111 GC patients at first diagnosis, 89 normal subjects, 48 superficial gastritis patients, and 28 postoperative GC patients by quantitative real-time PCR (qRT-PCR). Finally, we used the receiver operating characteristic (ROC) curve to analyze its diagnostic efficacy.

**Results:**

The expression of tRF-31-U5YKFN8DYDZDD has good stability and easy detection. tRF-31-U5YKFN8DYDZDD was highly expressed in tumor tissue, serum, and cell lines of GC, and the expression was significantly related to TNM stage, depth of tumor invasion, lymph node metastasis, and vascular invasion. The expression of serum tRF-31-U5YKFN8DYDZDD in the GC patients decreased after the operation (*P* = 0.0003). Combined with ROC curve analysis, tRF-31-U5YKFN8DYDZDD has better detection efficiency than conventional markers.

**Conclusions:**

The expressions of tRF-31-U5YKFN8DYDZDD in the tumor and paracancerous tissues, the serum of GC patients and healthy people, and the serum of GC patients before and after operation were different. tRF-31-U5YKFN8DYDZDD is not only a diagnostic biomarker of GC but also a predictor of poor prognosis.

## Highlights

- The expression of tRF-31-U5YKFN8DYDZDD was high in GC cells, tissues, and serum.
- tRF-31-U5YKFN8DYDZDD may serve as a potential biomarker for GC.
- High expression of tRF-31-U5YKFN8DYDZDD was associated with clinical prognostic factors.
- tRF-31-U5YKFN8DYDZDD combined with other markers can improve the diagnostic efficiency.

## Introduction

There are nearly one million new cases of gastric cancer (GC) in the world every year, and China accounts for about 40% of all, with the morbidity and mortality ranking among the top three malignancies in China (1, 2). GC is derived from the malignant transformation of gastric epithelial cells, and its pathological type is mainly adenocarcinoma (3). Because it is a hollow organ, the clinical symptoms in the early stage of malignant transformation are not obvious, mainly nausea, which is difficult to distinguish from diseases such as gastritis. Approximately 70% of the patients were already in the local progressive stage when diagnosed. It is of great clinical significance to improve the prognosis of patients with GC by early diagnosis and treatments (4). The early diagnosis of GC mainly depends on the pathology of gastroscopy, while the early screening mainly depends on the tumor biomarkers. Compared with gastroscopic diagnosis, hematological screening has the advantages of convenient, economical, and non-invasive detection and is easy to popularize (5). Carcinoembryonic antigen (CEA), carbohydrate antigen 199 (CA199), and carbohydrate antigen 724 (CA724) are relatively mature tumor biomarkers in clinical use at present, but their specificity and sensitivity are not high (6). Yu et al. demonstrated that the sensitivity of CEA in the diagnosis of gastric cancer is about 13–35%, while the specificity is only about 65%, and the CA199 is about 40 and 70%, respectively (7, 8). Therefore, new diagnostic biomarkers for GC are urgently needed in the clinic.

Non-coding RNA (ncRNAs) is the largest component of human transcriptome (9). There are many kinds of ncRNAs, which play important roles in the physiological and pathological processes of humans. Among them, the roles of microRNAs (miRNAs), long non-coding RNAs (lncRNAs), and circular RNAs (circRNAs) in the occurrence and development of cancers have been relatively thoroughly studied (10). Previous studies have shown that the ncRNAs above can be used as biomarkers for tumor diagnosis and prognosis evaluation (11, 12). Transfer RNA (tRNA) is also a kind of ubiquitously expressed and conservative ncRNAs. They account for about 10% of the entire cellular RNA and play a fundamental role in maintaining normal homeostasis, cell stress, stem cell differentiation, tumorigenesis, and cancer cell viability (13, 14).

The earliest reports of products derived from tRNAs can be traced back to the late 1970s, wherein fragments of tRNAs were observed in cancer patients (15). tRNA-derived small RNAs (tsRNAs) can be classified based on their cleavage sites of tRNA from which they are derived. tsRNAs are mainly divided into two subgroups: tRNA-derived fragments (tRFs) with lengths of 14–36 nt and tRNA halves (tiRNAs) with lengths of 30–40 nt (16, 17). tRFs are derived into 1-tRFs, 2-tRFs, 3-tRFs, 5-tRFs, and i-tRFs according to the different digesting positions of Angiogenin, Dicer, or other RNases on the mature tRNA or pre-tRNA, while tiRNAs including 5’- and 3’- fragments, named 5’-tiRNAs and 3’-tiRNAs, respectively (18, 19).

In recent years, as a new type of ncRNAs, the role of tsRNAs has gradually attracted people’s attention in cancers. There is a growing interest in whether tsRNAs can be used as a promising new biomarker. Numerous studies have indicated that tsRNAs may be potential biomarkers in breast cancer (20–22), ovarian cancer (23), lung cancer (24), prostate cancer (25–27), colorectal cancer (28, 29), renal cell carcinoma (30, 31), and others (18, 32). Huang Y et al. demonstrated that the expression of tDR-7816 could promote the occurrence of early non-triple-negative breast cancer and has been proved to be a biomarker for the diagnosis (33). Pekarsky et al. identified two new tiRNAs, ts-4521 and ts-3676, which were downregulated in lung cancer and chronic lymphocytic leukemia, exhibiting antitumor functions (34). In digestive tract tumors, 16 tRFs were identified as being significantly changed in colon cancer and paracancerous tissues (35). In GC, tRF-3019a regulates cell proliferation, migration, and invasion by targeting FBXO47, which may be a potential diagnosis biomarker (36). tRF-3017A was highly expressed in tissues and cell lines of GC and was positively correlated with lymph node metastasis. It may be that tRF-3017A promotes the migration and invasion of GC cells by silencing the tumor suppressor NELL2 (37). In the previous study, our team also found that serum hsa_tsr016141 has good stability and specificity and could be used for dynamic monitoring of patients with GC (38).

Based on the previous studies, we further explored the clinical significance of tsRNAs in GC. In this study, high-throughput sequencing was used to screen the high expression of tsRNAs in GC tissues, including tRF-31-U5YKFN8DYDZDD. The expression of serum tRF-31-U5YKFN8DYDZDD in the patients with GC diagnosed for the first time was detected, and the correlations with clinicopathological features were analyzed. Then, we evaluated the diagnostic efficacy of tRF-31-U5YKFN8DYDZDD in GC by receiver operating characteristic (ROC) analysis in an attempt to provide a novel biomarker.

## Materials and Methods

### Tissue Specimens and Serum Samples

In this study, the collections of serum and tissue samples of patients who signed informed consent were approved by the Ethics Committee of the Affiliated Hospital of Nantong University (approval No. 2018-L055). From 2016 to 2020, we collected sera of GC from 111 patients with newly diagnosed and 28 postoperative patients. We also collected sera from 89 healthy volunteers and 48 patients with gastritis. With the assistance of the department of gastrointestinal surgery and pathology of our hospital, we accumulated 24 pairs of GC and paracancerous tissues (T_1-4_N_1-0_M_0_, stage I–III). All the patients of GC were diagnosed by two different pathologists, and the patients did not receive neoadjuvant radiotherapy and chemotherapy. The paracancerous tissue, which had a distance of 3 cm from the tumor tissue, obtained from GC patients was confirmed to be free of tumor infiltration using H&E staining. After resection, the samples were put into the RNA fixator Bioteke (Nantong, China) immediately and stored in the refrigerator at −80°C.

### High-Throughput Sequencing

The total RNA or purified sRNA fragment of the sample was extracted, ligated at the 3’ end and 5’ end successively, reverse transcribed into cDNA, and then amplified by PCR. Then cut the glue to recover the target fragment library, and the qualified library was sequenced on Agilent 2100 Bioanalyzer (Agilent, USA). The raw reads obtained from Illumina HiSeqTM2500 (Illumina, USA) sequencing were filtered firstly, including removing the connectors at both ends of the reads, removing the reads with fragment length <15 nt, low-quality reads, etc., and obtaining the clean reads after preliminary filtering of the data. The whole-genome reads distribution map was obtained by comparing clean reads with the reference genome, and clean reads were classified and annotated by ncRNAs. The expression quantity calculation, expression clustering, and the difference among samples were carried out on the identified tRFs. tRFs were defined as the differentially expressed tRFs when log2FC > 1 or < −1 and Q value <0.05 using the DESeq2.0 algorithm.

### Cell Culture

The human GC cell lines MKN-1, MKN-45, AGS, BGC-823, MGC803, HGC-27, and SGC-7901 and normal gastric mucosal epithelial cell line GES-1 were purchased from the Shanghai Institutes for Biological Sciences, China Academy of Science (Shanghai, China). All cell lines were cultured with RPMI 1640 medium (REF.10-013-CV, Corning, Manassas, VA, USA) containing 10% fetal bovine serum (REF.10100-147, FBS, Gibco, Grand Island, NY, USA) and 100 U/ml penicillin-streptomycin mixture (REF.15140-122, GibCo BRL, Grand Island, NY, USA) in an atmosphere containing 5% CO2 at 37°C (Thermo, Waltham, MA, USA).

### Total RNA Extraction and cDNA Synthesis

Serum total RNA was extracted by Total RNA Pure and Isolation Kit with Spin Column (39–41) (Cat.RP4002, BioTeke, Beijing, China), while the tissue and cell total RNA was extracted using TRIzol reagent (Cat.15596018, Invitrogen, Karlsruhe, Germany). cDNA was amplified by Revert Aid RT Reverse Transcription Kit (Cat.K1622, Thermo Fisher Scientific, USA) at 42°C for 1 h and inactivated at 70°C for 5 min. The reverse transcription system was 10 µl. All steps were performed following the manufacturer’s instructions.

### qRT -PCR

All qRT-PCR assays were performed with the FastStart Universal SYBR Green Master Mix (Cat.Q711-02, Roche, Mannheim, Germany) on the QuantStudio 5 (Thermo, Waltham, MA, USA) for a total value of 20 μl. The reaction system included 10 µl of SYBR Green I Mix, 5 µl of cDNA, 1 µl of primer, and 3 µl of enzyme-free Water. To quantify the amount of tRFs, cDNA was synthesized from 500 ng of RNA. RNU6B (U6) was used as an internal control. All primers used in this study were synthesized by RiboBio Corporation (Suzhou, China). After the reaction, the 2^−ΔΔCT^ method was used to analyze the data results of relative expression level, and the ΔΔCt value was presented as the difference between the experimental group (Ct_(target)_ – Ct_(reference)_) and the control group (Ct_(target)_ – Ct_(reference)_). The relative expression level of each sample was divided by the mean of the expression levels of the references.

### Nuclear and Cytoplasmic RNA Separation Assay

The nuclear and cytoplasmic RNA was isolated from MKN-45 and HGC-27 cells using a PARIS™ Kit (Cat.AM1921, Thermo Fisher Scientific, USA) following the manufacturer’s instructions and subjected to qRT-PCR analysis. Up to 5 × 10^6^ GC cells were digested by trypsin and collected in a small centrifuge tube for the next steps. The experimental procedures have been provided in our previous study (42). The samples were tested by the RNA quality inspection before the next experiments.

### Actinomycin D Assay

The concentration of actinomycin D was 2.5 µg/ml (43, 44). The time points of total RNA extraction were 0, 2, 4, 8, 12, and 24 h after treatment with actinomycin D, respectively.

### Statistical Analysis

All data were analyzed by SPSS version 20.0 (IBM SPSS Statistics, Chicago, USA), GraphPad Prism v8.0 (Graphpad Software, La Jolla, CA, USA). The scatter plot drawn according to –△△Ct and paired t-test was used to describe the relative expression of tRF-31-U5YKFN8DYDZDD in preoperative *vs* postoperative and GC *vs* paracancerous tissues. Two-sided unpaired test was adopted for the comparison of two independent samples, while one-way analysis of variance was used to compare multiple independent samples. The relative expression of tRF-31-U5YKFN8DYDZDD and clinicopathological parameters was analyzed by chi-square test. The cutoff value of serum tRF-31-U5YKFN8DYDZDD expression to dichotomize as low and high was the median of relative expression. If the expression level was higher than the median, it was considered to be high expression of tRF-31-U5YKFN8DYDZDD; on the contrary, it was recognized as low expression (21, 38). For the analysis of survival data, Kaplan–Meier curves were constructed, and the log-rank test was performed. ROC curve and area under the curve (AUC) were used to evaluate the diagnostic performance of tRF-31-U5YKFN8DYDZDD in GC. Before plotting the ROC curve, we performed binomial logistic regression. Multivariate analysis was performed using Cox’s proportional hazards model. The risk ratio and its 95% confidence interval (CI) were recorded for each marker. All experiments were repeated independently at least three times. Mean value ± standard deviation (SD) was used to list Data. P < 0.05 was considered statistically significant.

## Results

### Expression of tRFs in GC Tissues and Cell Lines

**Figure 1:**
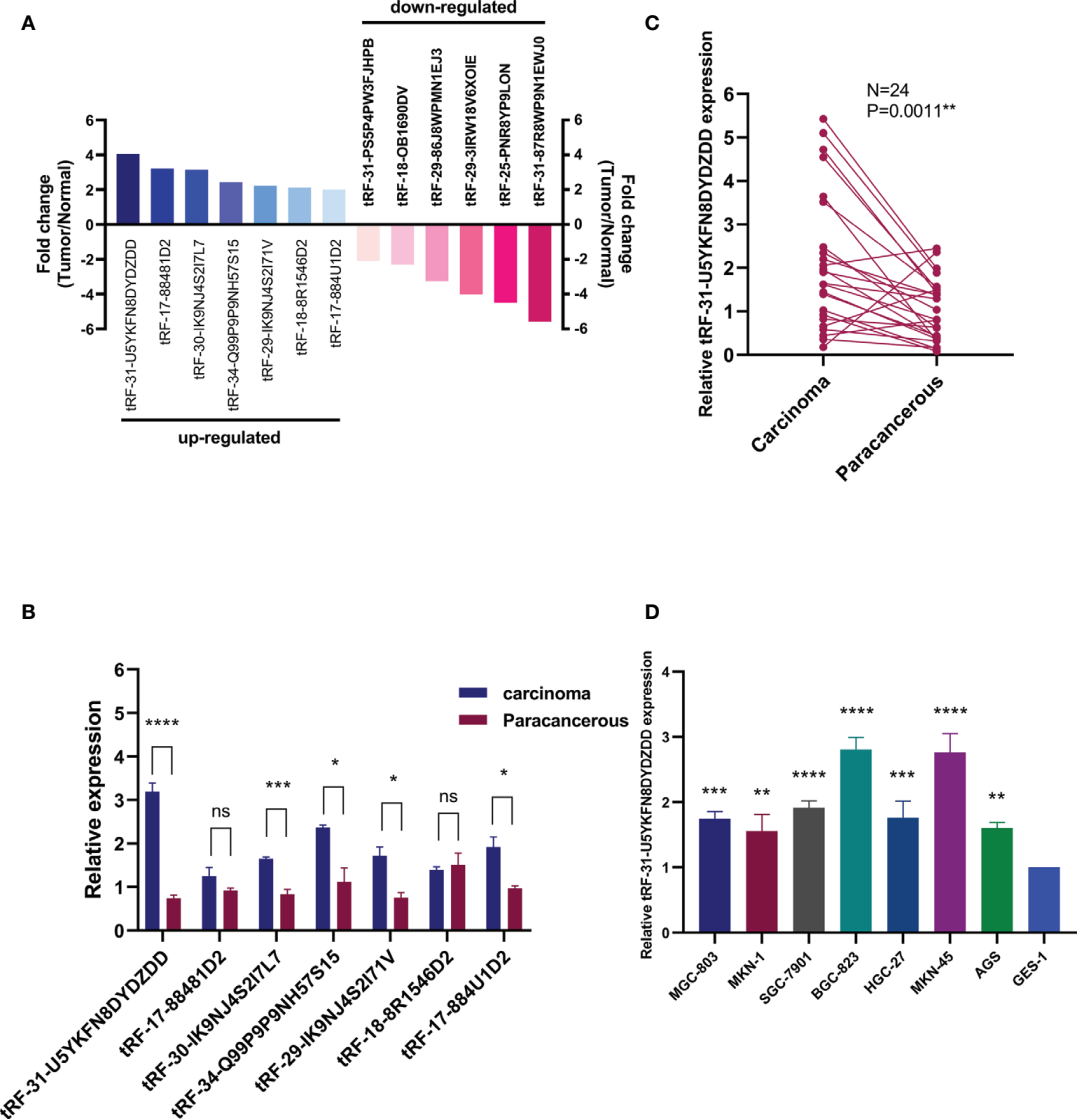
*Expression of tRFs in GC tissues and cell lines.***(A)** Differential expression results of tRFs-seq for GC *vs.* matched paracancerous tissues, including seven upregulated and six downregulated (fold change > 2.0, *P* < 0.05)**. (B)** The expression levels of tRFs in GC tissues and their paired adjacent paracancerous tissues. **(C)** Expression of tRF-31-U5YKFN8DYDZDD was quantified by qRT-PCR in tissues, and its expression is normalized by U6 RNA in each sample. **(D)** Expression of tRF-31-U5YKFN8DYDZDD in GC cells and normal gastric mucosal epithelial cell line (GES-1). U6 was used for normalization. *Indicated statistical significance (****P < 0.0001, ***P < 0.001, **P < 0.01, *P < 0.05); NS, no significance.

To study the expression of tRFs in GC, we used a high-throughput sequencing technique to determine differential expression of tRFs in three pairs of GC patients and matched paracancerous specimens. There were about 5,512 different expression tRFs detected in total. According to the tRF-Seq data, we identified the tRFs between the two groups, of which seven were upregulated (fold change >2.0, *P* < 0.05) and six were downregulated (fold change <−2.0, *P* < 0.05) in GC tissues relative to paracancerous tissues (**Figure 1A**). According to the results of high-throughput sequencing, we verified the expression of tRF in another three pairs of GC and paracancerous tissues (**Figure 1B**) and found that the results were basically consistent with the results of sequencing. The difference in the expression of tRF-31-U5YKFN8DYDZDD between GC and paracancerous tissues was the most significant, which is the key molecule of this study. To validate the results of tRF-Seq data, we further collected 24 pairs of GC samples and detected the expression of tRF-31-U5YKFN8DYDZDD. Intriguingly, significantly higher levels of tRF-31-U5YKFN8DYDZDD were detected in carcinomas than paracancerous specimens (*P* = 0.0011, **Figure 1C**). Meanwhile, we detected the expression of tRF-31-U5YKFN8DYDZDD in different GC cell lines and found that tRF-31-U5YKFN8DYDZDD was significantly increased in GC cells as compared to normal gastric mucosal epithelial cell line GES-1 (*P* < 0.01, **Figure 1D**).

### tRF-31-U5YKFN8DYDZDD Is a Type of i-tRF

**Figure 2:**
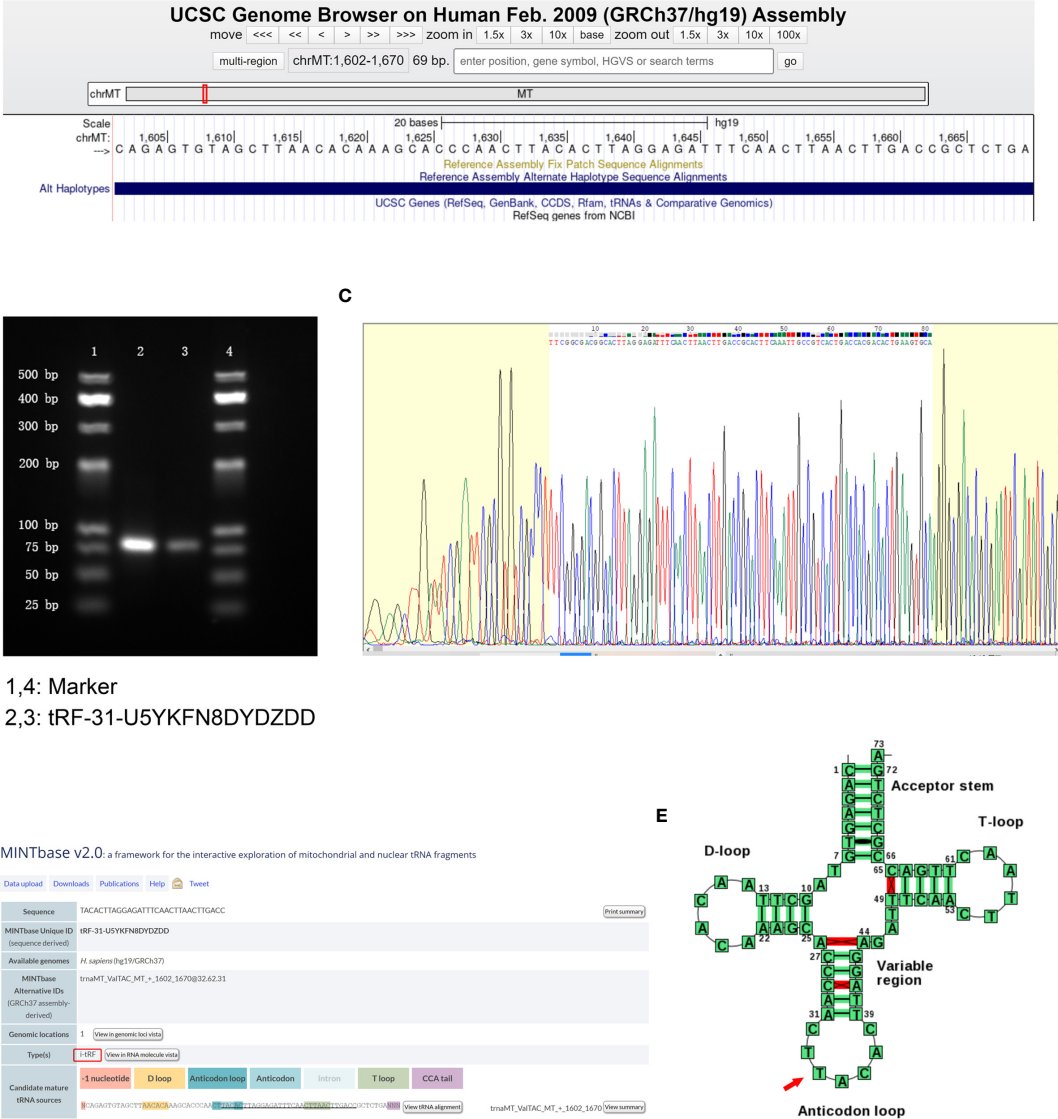
*tRF-31-U5YKFN8DYDZDD is a type of i-tRF.***(A)** tRF-31-U5YKFN8DYDZDD is located on ChrMT with coordinates of 1,602–1,670 and the length of 69 bp using the UCSC Genome Browser database (human genome build GRCh37/hg19). Search the relevant database for details. **(B)** The product of qRT-PCR was run on 2.5% agarose gel showing a single electrophoresis band about 80 bp in size. **(C)** The product of qRT-PCR was confirmed by Sanger sequencing, which contained the full-length sequence of tRF-31-U5YKFN8DYDZDD. **(D)** tRF-31-U5YKFN8DYDZDD was an i-tRF with a length of 31 nt (5’- TACACTTAGGAGATTTCAACTTAACTTGACC-3’) in the MINTbase v2.0. **(E)** The cleavage site of tRF-31-U5YKFN8DYDZDD was located above the anticodon loop (CTTACAC) in Transfer RNA Database.

According to the human genome build (GRCh37/hg19) from the UCSC Genome Browser database (https://genome.ucsc.edu/), tRF-31-U5YKFN8DYDZDD was mapped to ChrMT with coordinates of 1,602–1,670, and the length was 69 bp (**Figure 2A**). To verify the accuracy of the product of qRT-PCR, we detected the amplification procedure by agarose gel electrophoresis (AGE) assay and showed a single electrophoresis band about 80 bp in size (**Figure 2B**). After recovering and cloning, they were confirmed by Sanger sequencing that the product contained the full-length sequence of tRF-31-U5YKFN8DYDZDD (**Figure 2C**). In the MINTbase v2.0 (http://cm.jefferson.edu/MINTbase/), tRF-31-U5YKFN8DYDZDD was an i-tRF with a length of 31nt (5’- TACACTTAGGAGATTTCAACTTAACTTGACC -3’) (**Figure 2D**). According to the basic information of tRF-31-U5YKFN8DYDZDD in Transfer RNA Database (http://trna.bioinf.uni-leipzig.de/), the cleavage site was located on the anticodon loop (CTTACAC) (**Figure 2E**).

### Characteristics of the tRF-31-U5YKFN8DYDZDD as a Biomarker for GC

**Figure 3:**
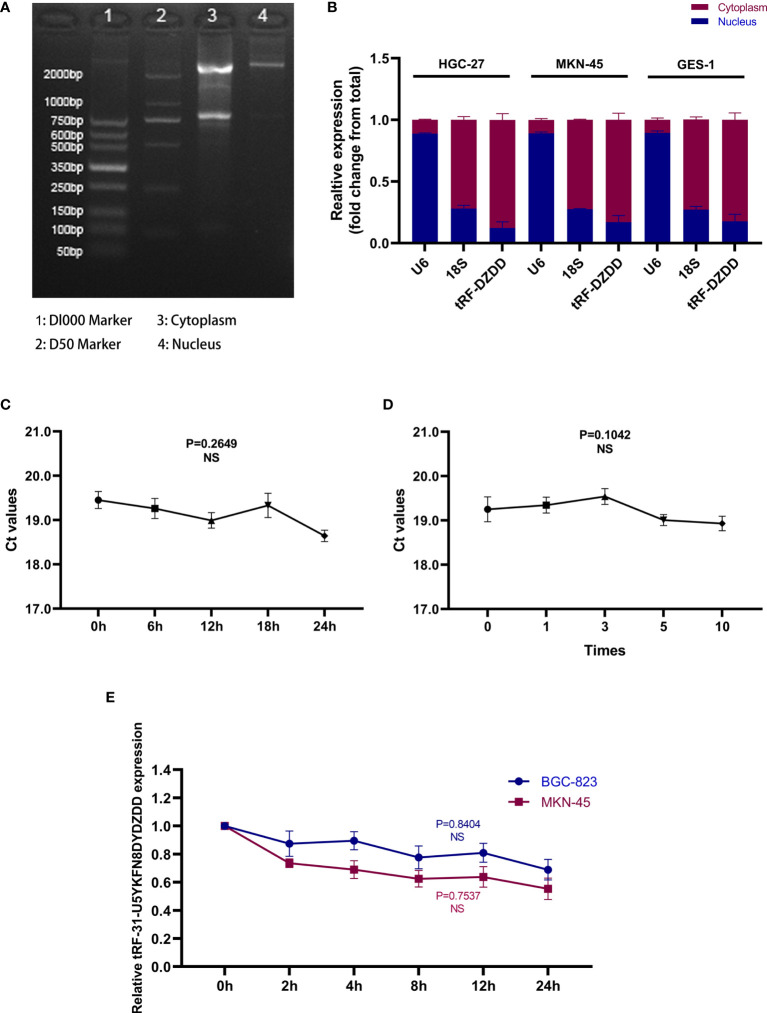
*Characteristics of the tRF-31-U5YKFN8DYDZDD as a biomarker for GC.****(*A*)*** The RNA quality inspection of RNA Separation Assay of HGC-27 cells was detected by agarose gel electrophoresis. **(B)** Detection of tRF-31-U5YKFN8DYDZDD location in HGC-27, MKN-45, and GES-1 cell lines by Nuclear and Cytoplasmic RNA Separation Assay. **(C, D)** The good stability and repeatability of tRF-31-U5YKFN8DYDZDD were confirmed by qRT-PCR. **(E)** qRT-PCR for abundance of tRF-31-U5YKFN8DYDZDD in in BGC-823 and MKN-45 cell lines treated with Actinomycin D at the indicated time point.

To further clarify the possibility of tRF-31-U5YKFN8DYDZDD as a biomarker for GC, we studied its characteristics. First of all, we used the Nuclear and Cytoplasmic RNA Separation Assay to test the expression of tRF-31-U5YKFN8DYDZDD in HGC-27 and MKN-45cell lines. The RNA quality inspection of RNA Separation Assay is shown in **Figure 3A**. It is mainly located in the cytoplasm and can be further secreted into the extracellular fluid (**Figure 3B**). The expression trend was consistent with that in cell lines and tissues. It can be inferred that tRF-31-U5YKFN8DYDZDD is secreted from the cytoplasm to the extracellular fluid, and its expression can be determined directly. Next, we placed the mixed serum samples at room temperature for 0, 6, 12, 18, and 24 h and repeated freeze-thaw for 0, 1, 3, 5, and 10 times. There is no statistical difference in the relative expression of tRF-31-U5YKFN8DYDZDD in the above two experiments (P > 0.05), which indicated that its detection would not be easily affected (**Figures 3C, D**). Meanwhile, to explore whether the detection of tRF-31-U5YKFN8DYDZDD can be applied in clinical practice, we conducted the repeatability of its detection methods. We selected mixed serum for precision determination of tRF-31-U5YKFN8DYDZDD and found that the coefficient of variation (CV) performed well. The results showed that the CV of tRF-31-U5YKFN8DYDZDD in intra-assay was 3.76% and in the inter-assay it was 3.19% (**Table 1**). Finally, the stable expression of tRF-31-U5YKFN8DYDZDD was further verified by actinomycin D assay. After treatment with actinomycin D for 24 h, the expression of tRF-31-U5YKFN8DYDZDD in BGC-823 and MKN-45 cell lines did not decrease significantly (**Figure 3E**). tRF-31-U5YKFN8DYDZDD was stable with the effect of actinomycin D and had a longer half-life. The above experiments can preliminarily determine that tRF-31-U5YKFN8DYDZDD can be detected as a biomarker in GC serum, and the detection method of tRF-31-U5YKFN8DYDZDD had high stability and repeatability.

**Table 1:** The intra-assay coefficient of variation and the inter-assay coefficient of variation of tRF-31-U5YKFN8DYDZDD.

	tRF-31-U5YKFN8DYDZDD	U6
**Intra-assay CV, %**	3.76	1.61
**Inter-assay CV, %**	3.19	2.35

CV (coefficient of variance) = SD/Mean × 100%.

### Expression of tRF-31-U5YKFN8DYDZDD in GC Serum and the Correlation With the Clinicopathological Parameter

**Figure 4:**
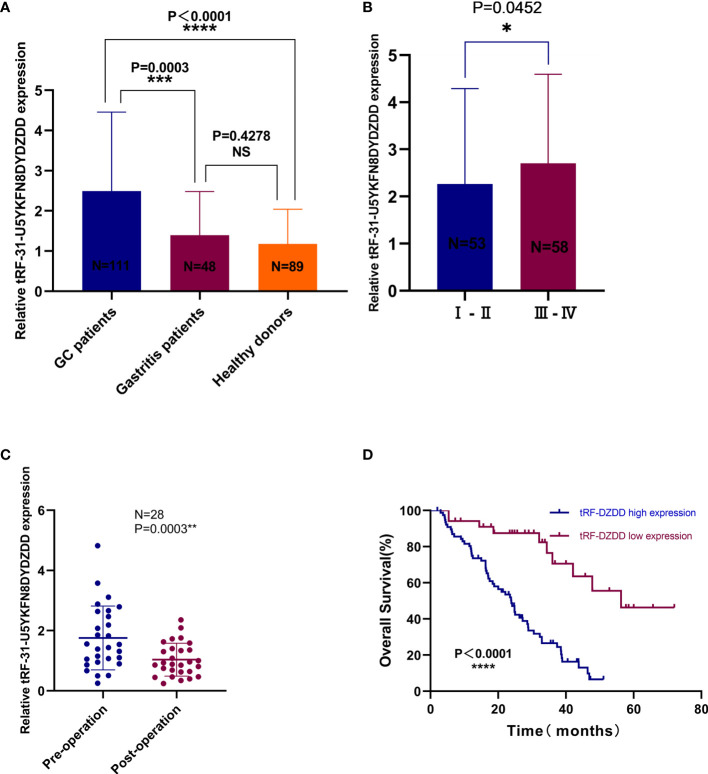
*Expression of tRF-31-U5YKFN8DYDZDD in GC serum.***(A)** The expression of tRF-31-U5YKFN8DYDZDD expression levels between GC (n = 111), gastritis patients (n = 48), and healthy volunteers (n = 89). **(B)** The expression levels of tRF-31-U5YKFN8DYDZDD in different pathologic stages of GC patients (stage I–II, n = 53; stage III–IV, n = 58). **(C)** Paired comparison of tRF-31-U5YKFN8DYDZDD in 28 pairs of preoperative and postoperative patients’ serums was determined using qRT-PCR. **(D)** Kaplan–Meier survival curves for 111 GC patients according to tRF-31-U5YKFN8DYDZDD expression status (log-rank test, *P* < 0.001).

tRF-31-U5YKFN8DYDZDD already has the basic characteristics as a biomarker, and the specificity of tRF-31-U5YKFN8DYDZDD in GC diagnosis and its correlation with clinicopathological data would be further studied. Firstly, the expressions of tRF-31-U5YKFN8DYDZDD in 111 GC patients, 48 gastritis patients, and 89 healthy donors’ serum samples were evaluated by qRT-PCR (**Figure 4A**). We found the expression of tRF-31-U5YKFN8DYDZDD in serum from 111 GC patients was significantly increased as compared to healthy controls (*P* < 0.0001) and gastritis patients (*P* = 0.0003). Chi-square test was used to analyze the clinicopathologic parameter of 111 GC patients to further explore the potential clinical value of tRF-31-U5YKFN8DYDZDD expression level and clinicopathologic features (**Table 2**). As shown, we found that higher tRF-31-U5YKFN8DYDZDD expression was significantly associated with depth of tumor invasion (*P* = 0.016), lymph node metastasis (*P* = 0.010), higher TNM stage (*P* = 0.003), and positive vascular invasion (*P* = 0.033), but no significant relationship with age, gender, differentiation grade, tumor size, Lauren classification, nerve invasion, and the expression of C-erbB-2, CEA, CA199, CA724. Secondly, increased tRF-31-U5YKFN8DYDZDD expression in GC was significantly correlated with in different stage GC (**Figure 4B**, *P* = 0.0452), which is consistent with the results in **Table 2**. Besides, the expression of tRF-31-U5YKFN8DYDZD in 28 pairs of preoperative and postoperative GC specimens was confirmed using qRT-PCR assay (**Figure 4C**). The statistical analysis results showed that the tRF-31-U5YKFN8DYDZDD expression had a close correlation with tumor burden (*P* = 0.0003). Kaplan–Meier analysis revealed that high tRF-31-U5YKFN8DYDZDD expression was significantly correlated with shorter overall survival (*P* < 0.0001, log-rank test; **Figure 4D**). Furthermore, multivariate Cox regression analysis indicated that tRF-31-U5YKFN8DYDZDD expression was an independent prognostic factor (HR = 4.179, 95% CI 2.143–8.149, P < 0.001; **Table 3**). Given that no studies have reported this novel tRNA derivative, we reasoned that tRF-31-U5YKFN8DYDZDD may act as a biomarker for the diagnosis of GC, to judge the tumor load, and as a tumor correlation factor associated with poor prognosis.

**Table 2:** The association between tRF-31-U5YKFN8DYDZDD expression and clinicopathologic parameters in 111 GC specimens.

Parameters	Total	tRf-DZDD expression		*P* value
		Low (＜median)	High (≥median)	
**Age**				0.504
<60	37	12	25	
≥60	74	19	55	
**Gender**				0.518
Male	70	18	52	
Female	41	13	28	
**Differentiation grade**				0.527
Well-moderate	49	12	37	
Poor-undifferentiation	62	19	43	
**Tumor size**				0.078
<5 cm	70	24	46	
≥5 cm	41	7	34	
**Depth of invasion**				0.016*
T1–T2	43	18	25	
T3–T4	68	13	55	
**Lymph node metastasis**				0.010*
Negative	46	19	27	
Positive	65	12	53	
**TNM stage**				0.003**
I–II	53	22	31	
III–IV	58	9	49	
**Lauren classification**				0.907
Intestinal type	32	8	24	
Diffuse type	34	10	24	
Mixed type	45	13	32	
**Nerve invasion**				0.668
Negative	67	20	47	
Positive	44	11	33	
**Vascular invasion**				0.033*
Negative	46	18	28	
Positive	65	13	52	
**C-erbB-2**				0.113
Negative	102	26	76	
Positive	9	5	4	
**CEA**				0.833
Negative (<5 ng/ml)	48	14	34	
Positive (≥5 ng/ml)	63	17	46	
**CA199**				0.385
Negative (<37 U/ml)	71	22	49	
Positive (≥37 U/ml)	40	9	31	
**CA724**				0.831
Negative (<10 U/ml)	46	19	46	
Positive (≥10 U/ml)	65	12	34	

Statistical analyses were performed by the Pearson χ^2^ test.

*P < 0.05, **P < 0.01 was considered significant.

**Table 3:** Multivariate Cox regression analysis of tRF-31-U5YKFN8DYDZDD and clinical variables predicting survival from 111 GC specimens.

Parameter	Relative ratio	95% CI	*P* Value
**Age** (<60 *vs.* ≥60)	0.608	0.355–1.042	.070
**Gender** (Male *vs.* Female)	1.204	0.720–2.015	.479
**Differentiation grade** (Well-moderate *vs.* Poor-undifferentiation)	0.806	0.449–1.447	.470
**Tumor size** (<5 *vs.* ≥5 cm)	0.814	0.471–1.405	.459
**Depth of invasion** (T1–T2 *vs.* T3–T4)	1.429	1.032–1.979	.032*
**Lymph node metastasis** (Negative *vs.* Positive)	1.459	0.671–3.171	.340
**TNM stage** (I–II *vs.* III–IV)	0.399	0.162–.984	.046*
**Lauren classification** (Intestinal type *vs.* Diffuse type *vs.* Mixed type)	0.897	0.457–1.760	.752
**Nerve invasion** (Negative *vs.* Positive)	1.719	0.915–3.230	.092
**Vascular invasion** (Negative *vs.* Positive)	0.574	0.308–1.068	.080
**C-erbB-2** (Negative *vs.* Positive)	1.329	0.430–4.107	.621
**CEA** (<5 *vs.* ≥5 ng/ml)	0.690	0.406–1.173	.171
**CA199** (<37 *vs.* ≥37 U/ml)	0.865	0.507–1.476	.595
**CA724** (<10 *vs.* ≥10 U/ml)	1.381	0.813–2.347	.232
**tRF-31-U5YKFN8DYDZDD** (Negative *vs.* Positive)	4.179	2.143–8.149	.001**

*P < 0.05, **P < 0.01 was considered significant.

### Evaluation of the Diagnostic Accuracy of Serum tRF-31-U5YKFN8DYDZDD and Combined Diagnostic Model in GC

**Figure 5:**
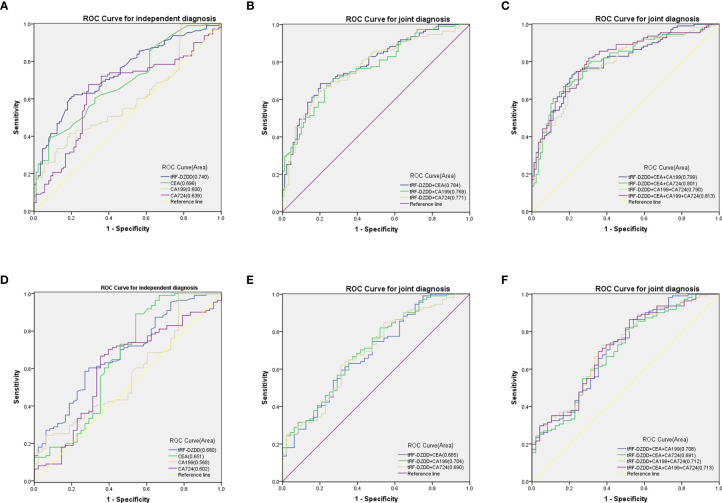
*Evaluation of the diagnostic accuracy of serum tRF-31-U5YKFN8DYDZDD and combined diagnostic model in GC.***(A–C)** ROC curves for GC diagnosis using serum tRF-31-U5YKFN8DYDZDD, CEA, CA199, and CA724 between GC patients and healthy controls. **(D–F)** ROC curves for GC diagnosis using serum tRF-31-U5YKFN8DYDZDD, CEA, CA199, and CA724 between GC and gastritis patients. **(B, C, E, F)** Combined diagnostic efficacy of serum tRF-31-U5YKFN8DYDZDD, CEA, CA199, and CA724 exerted the best diagnostic efficacy in distinguishing GC patients and non-malignant disease.

Compared with mature GC biomarkers such as CEA, CA199, and CA724, the diagnostic efficiency of tRF-31-U5YKFN8DYDZDD is the key factor to evaluate. The ROC curve showed that the AUC of tRF-31-U5YKFN8DYDZDD was 0.740 (95% CI: 0.6720–0.808), which was higher than 0.696 of CEA (95% CI: 0.624–0.768), 0.600 of CA199 (95% CI: 0.521–0.678), and 0.639 of CA724 (95% CI: 0.561–0.718) (**Figure 5A**). And the level of tRF-31-U5YKFN8DYDZDD presented a 60.36% sensitivity (SEN) and 80.90% specificity (SPE) in separating GC patients from healthy controls. As for CEA, CA199, and CA724, the SEN was 57.66, 36.04, and 42.34%; and the SPE was 67.42, 82.02, and 74.16%, respectively (**Table 4**). Using the healthy group as the control, some diagnostic test evaluation indicators were also calculated in the two-combination group, three-combination group, or four-combination group to assess their SEN, SPE, overall accuracy (ACCU), positive predictive value (PPV), and negative predictive value (NPV). The efficacy of joint diagnosis of AUC increased to 0.783 after the combination of tRF-31-U5YKFN8DYDZDD and CEA, 0.769 after combining with CA199, and 0.771 after combining with CA724 (**Figure 5B**). As shown in **Figures 5B, C**, the model combining four indicators yielded a good diagnostic efficacy for GC patients with an AUC of 0.813 (95% CI: 0.754–0.873), which was higher than that of the two-/three-combination group or either of the four indicators alone. Interestingly, it was found that the SEN, ACCU, and NPV values of the four-combination group were 81.98, 76.50, and 75.61%, respectively, which were the highest of the 11 groups (**Table 4**). The above findings indicate that tRF-31-U5YKFN8DYDZDD may be a potential biomarker of GC and, combined with other tumor markers, can improve the diagnostic efficiency.

**Table 4:** Use the expression levels of tRF-31-U5YKFN8DYDZDD, CEA, CA199, and CA724 to distinguish GC patients from healthy donors.

	SEN, %	SPE, %	ACCU, %	PPV, %	NPV, %
**tRF-DZDD**	60.36 (67/111)	80.90 (72/89)	69.50 (139/200)	79.76 (67/84)	62.07 (72/116)
**CEA**	57.66 (64/111)	67.42 (60/89)	62.00 (124/200)	68.82 (64/93)	56.07 (60/107)
**CA199**	36.04 (40/111)	82.02 (73/89)	56.50 (113/200)	71.43 (40/56)	50.69 (73/144)
**CA724**	42.34 (47/111)	74.16 (66/89)	56.50 (113/200)	67.14 (47/70)	50.77 (66/130)
**tRF-DZDD+CEA**	68.47 (76/111)	79.78 (71/89)	73.50 (147/200)	80.85 (76/94)	66.98 (71/106)
**tRF-DZDD+CA199**	71.17 (79/111)	73.03 (65/89)	72.00 (144/200)	76.70 (79/103)	67.01 (65/97)
**tRF-DZDD+CA724**	63.96 (71/111)	80.90 (72/89)	71.50 (143/200)	80.68 (71/88)	64.29 (72/112)
**tRF-DZDD+CEA+CA199**	72.97 (81/111)	78.65 (70/89)	75.50 (151/200)	81.00 (81/100)	70.00 (70/100)
**tRF-DZDD+CEA+CA724**	66.67 (74/111)	83.15 (74/89)	74.00 (148/200)	83.15 (74/89)	66.67 (74/111)
**tRF-DZDD+ CA199+CA724**	74.77 (83/111)	74.16 (66/89)	74.50 (149/200)	78.30 (83/106)	70.21 (66/94)
**tRF-DZDD+CEA+ CA199+CA724**	81.98 (91/111)	69.66 (62/89)	76.50 (153/200)	77.12 (91/118)	75.61 (62/82)

SEN, sensitivity; SPE, specificity; ACCU, overall accuracy; PPV, positive predictive value; NPV, negative predictive value.

In serum samples, the expression of tRF-31-U5YKFN8DYDZDD is also different in GC and gastritis, so it is very important to further study the efficacy of tRF-31-U5YKFN8DYDZDD in the distinction between GC and gastritis. We compared the four indicators and found that the related sensitivity and specificity of tRF-31-U5YKFN8DYDZD were 58.56 and 72.92%, respectively, with the AUC of 0.680 (95% CI:0.591–0.769) *vs.* 0.651 for CEA (95% CI: 0.547–0.755) *vs.* 0.568 for CA199 (95% CI: 0.471–0.666) and 0.602 for CA724 (95% CI: 0.503–0.701) (**Table 5** and **Figure 5C**). Similar to the results of tRF-31-U5YKFN8DYDZD expression in normal serum, the combination of tRF-31-U5YKFN8DYDZDD and other tumor markers was the highest value for diagnosis between GC and gastritis. Both **Figures 5D–F** and **Table 5** show that the combined detection of serum tRF-31-U5YKFN8DYDZDD, CEA, CA199, and CA724 is superior to any of the biomarkers detected separately in the diagnosis of GC patients (AUC = 0.713). Besides, the combination of tRF-31-U5YKFN8DYDZDD, CEA, CA199, and CA724 could improve the diagnostic SEN (86.49%), ACCU (74.84%), and NPV (60.53%), which were better than these in the two-/three-combination group or any of the four indicators alone. All these results suggest that tRF-31-U5YKFN8DYDZD seemed better than CEA, CA199, and CA724 in terms of the diagnostic value for GC.

**Table 5:** Use the expression levels of tRF-31-U5YKFN8DYDZDD, CEA, CA199, and CA724 to distinguish GC patients from gastritis patients.

	SEN, %	SPE, %	ACCU, %	PPV, %	NPV, %
**tRF-DZDD**	58.56 (65/111)	72.92 (35/48)	62.89 (100/159)	83.33 (65/78)	43.21 (35/81)
**CEA**	57.66 (64/111)	62.50 (30/48)	59.12 (94/159)	78.05 (64/82)	38.96 (30/77)
**CA199**	36.04 (40/111)	70.83 (34/48)	46.54 (74/159)	74.07 (40/54)	32.38 (34/105)
**CA724**	42.34 (47/111)	68.75 (33/48)	50.31 (/15980)	75.81 (47/62)	34.02 (33/97)
**tRF-DZDD+CEA**	59.46 (66/111)	68.75 (33/48)	62.26 (99/159)	81.48 (66/81)	42.31 (33/78)
**tRF-DZDD+CA199**	81.98 (91/111)	47.92 (23/48)	71.70 (114/159)	78.45 (91/116)	53.49 (23/43)
**tRF-DZDD+CA724**	62.16 (69/111)	68.75 (33/48)	64.15 (102/159)	82.14 (69/84)	44.00 (33/75)
**tRF-DZDD+CEA+CA199**	86.49 (96/111)	45.83 (22/48)	74.21 (118/159)	78.69 (96/122)	59.46 (22/37)
**tRF-DZDD+CEA+CA724**	81.08 (90/111)	50.00 (24/48)	71.70 (114/159)	78.95 (90/114)	53.33 (24/45)
**tRF-DZDD+ CA199+CA724**	72.97 (81/111)	62.50 (30/48)	69.81 (111/159)	81.82 (81/99)	50.00 (30/60)
**tRF-DZDD+CEA+ CA199+CA724**	86.49 (96/111)	47.92 (23/48)	74.84 (119/159)	79.34 (96/121)	60.53 (23/38)

SEN, sensitivity; SPE, specificity; ACCU, overall accuracy; PPV, positive predictive value; NPV, negative predictive value.

### Target Prediction of tRF-31-U5YKFN8DYDZDD

**Figure 6:**
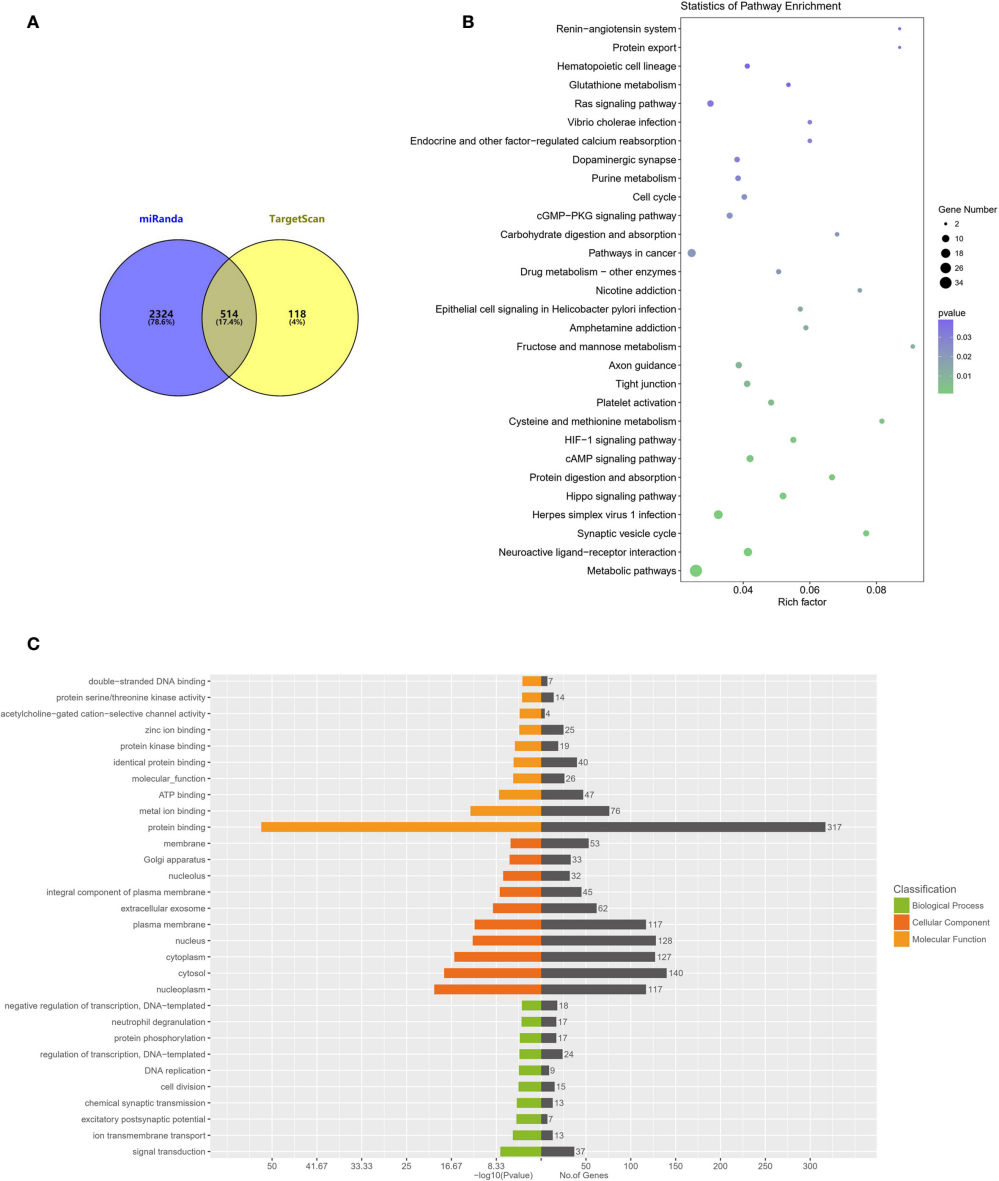
*GO and KEGG enrichment analyses of tRF-31-U5YKFN8DYDZDD target genes.***(A)** Schematic diagram of tRF-31-U5YKFN8DYDZDD target prediction. Venn diagram evaluated the overlapped genes between miRandan and TargetScan predictions. **(B)** Bubble chart of KEGG analysis of candidate tRF-31-U5YKFN8DYDZDD different target genes expressed of enriched pathways. **(C)** GO analysis of the tRF-31-U5YKFN8DYDZDD target genes enriched in biological process, cellular component, and molecular function.

We next want to investigate the molecular mechanism of tRF-31-U5YKFN8DYDZDD in cell biological behavior regulation. We predict downstream target genes of tRF-31-U5YKFN8DYDZDD using online databases. As shown in **Figure 6A**, overlapped between miRanda and TargetSca prediction tools were 514 potential target genes that are most likely to bind to tRF-31-U5YKFN8DYDZDD. Next, enrichment analysis of the KEGG signaling pathway suggested that cell cycle, pathways in cancer, and drug metabolism were significantly enriched in the signaling pathways (**Figure 6B**). GO functional enrichment analysis of the target genes indicated that tRF-31-U5YKFN8DYDZDD (**Figure 6C**) may have the potential role in signal transduction, cell division, and regulation of transcription. The mechanisms of tRF-31-U5YKFN8DYDZDD expression in cell biological behavior regulation in GC need to be further investigated.

## Discussion

As a common malignant tumor of the digestive tract, the incidence and mortality of GC are among the highest, which is mainly related to the late course of the disease and poor response to treatment (45). Although the prognosis of GC has improved with the continuous updating and development of surgical methods, chemotherapeutic drugs, and targeted drugs, the 5-year survival rate is still not high, and the incidence rate is stable without significant decline (46). In conclusion, improving the early diagnosis and treatment of GC is the key to improve the prognosis of patients. However, the specificity and sensitivity of clinical tumor biomarkers are low, so it is particularly urgent to look for new GC screening markers. The occurrence and development of GC is a multistage and multifactor process. Now the multiple genetic and epigenetic changes of coding genes in the complex regulatory interaction network have become the focus of oncology research, including GC (47, 48). With the development of microarray and RNA sequencing technology, more and more ncRNAs have been identified. Their roles and functions have also been gradually studied in depth. This paper focuses on the role of ncRNA in the early diagnosis and prognosis of GC.

Different from common ncRNAs, the function and study of tRNAs are not as thorough as microRNAs, lncRNAs, and circRNAs. tRNAs routinely play a role in translation, and tRNA(~72 nt) can be processed into smaller bioactive tRNA-derived fragments, ranging in size from 18 to 50 nt, which play a role in the occurrence and progression of tumors (15, 49). tRNAs can give rise to different types of tRNA-derived fragments, including tRFs with lengths of 14–36 nt and tiRNAs with lengths of 30–40 nt (50). tRNAs can also produce other kinds of small RNAs, in which the start or end position does not match the 5’ or 3’ end of the parent tRNA. These small RNAs are generally called i-tRFs, which belong to tRFs. The number of i-tRF is usually small compared with other types (14). The biological functions of tRFs include acting as a miRNA, regulating translation, regulating the expression and silencing of target genes, and participating in cellular stress response. The understanding of tRF-mediated cancer progression is still in the initial stage. Previous studies have shown that tRF is involved in various cellular stages, such as differentiation, proliferation and apoptosis, chromatin remodeling, RNA editing, and RNA splicing, which lead to cancer (17, 28). Fei Zhang et al. demonstrated that tRF-3019a enhanced cell proliferation, migration, and invasion by targeting FBXO47, and it might serve as a potential diagnostic biomarker for GC (36). Similar results were found that tRF-3017A might play an important role in promoting migration and invasion by silencing NELL2 in GC (37). In this study, tRFs were screened from GC tissues by high-throughput sequencing. The results showed that the expression of tRF-31-U5YKFN8DYDZDD in GC was significantly higher than that in normal control or cancer after *in vitro* verification in cell lines, serums, and tissues. After further analysis, it was found that tRF-31-U5YKFN8DYDZDD belonged to i-tRF. Its stable structure and high expression in body fluids make it relatively easy to detect, which makes it possible to become a new potential biomarker for tumors. Currently, most studies on tsRNAs have described the effects on proliferation, invasion, and migration of tumor cells and the mechanisms involved in hypoxia and epithelial to mesenchymal transformation (EMT) (10, 21, 51, 52). However, there are few studies on whether tsRNAs can be used as a biomarker for tumors (18, 24, 53). Therefore, this study explored the possibility of tRF-31-U5YKFN8DYDZDD as a tumor biomarker for GC, which was highly innovative.

In the present study, it was proved that the expression of tRF-31-U5YKFN8DYDZDD in GC tissues was significantly higher than that in paracancerous tissues, the expression in GC cell lines was also higher than that in normal gastric epithelial cells, and the expression in GC serum was significantly higher than that in normal physical examination population and gastritis patients, with statistically significant differences. The results of qRT-PCR detection were consistent with those of high-throughput sequencing, indicating that the high expression of tRF-31-U5YKFN8DYDZDD was closely related to tumorigenesis. After the detection of serum tRF-31-U5YKFN8DYDZDD expression in patients before and after the operation, it was found that the tumor load reduced and the expression of tRF decreased significantly after the operation, which can be used as an index for dynamic monitoring of tumor load, and may also be an important indication of tumor recurrence. Statistical analysis of large samples showed that the high expression of tRF-31-U5YKFN8DYDZDD was positively correlated with late-stage, deep tumor invasion, lymph node metastasis, and vascular invasion in patients with GC, and was a related factor of poor prognosis. The survival time of patients with high expression of tRF-31-U5YKFN8DYDZDD is significantly lower than that of patients with low expression, which is an independent prognostic factor. For the related experiments of the molecular characteristics of tRF-31-U5YKFN8DYDZDD itself, chromosome location, and PCR amplification sequencing, it is clear that it has the basic conditions to become a biomarker. Further ROC curve analysis showed that tRF-31-U5YKFN8DYDZDD had high sensitivity and specificity, which was superior to conventional markers such as CEA in differentiating diagnosis of benign and malignant gastric tumors. What is more exciting is that the combined diagnosis of tRF-31-U5YKFN8DYDZDD with CEA, CA199, and CA724 has more diagnostic potency and good clinical application potential.

High-throughput sequencing revealed tsRNA signatures in cancers, indicating that like microRNAs, tsRNAs may have an oncogenic or tumor-suppressor function in tumors (34, 54). In the study of cellular function, it has been found that tRF-33-P4R8YP9LON4VDP could proliferate GC cells *in vitro* and might be a potential site for targeted therapy (55). In breast cancer research, it was also found that the runt-related transcription factor 1 (Runx 1) can reverse the excessive proliferation of tumor cells induced by ts-112 (56). tsRNA-26576 can not only promote the proliferation of tumor cells but also promote invasion and migration in breast cancer (57). In this study, we demonstrated the existence of tRF-31-U5YKFN8DYDZDD in GC cells, tissues, and serum. Moreover, tRF-31-U5YKFN8DYDZDD in GC patients have significantly higher tsRNA levels than that in healthy donors, indicating their great potential as a novel “liquid biopsy” biomarker for GC diagnosis. Notably, it was statistically found that the high expression of tRF-31-U5YKFN8DYDZDD was associated with late-stage, deep tumor invasion, lymph node metastasis, and vascular invasion, and these indicators were closely related to the invasion and migration ability of tumor cells. Of course, the proliferation ability of GC cells cannot be ignored. These laid a theoretical foundation for our further study of the functional role of tRF-31-U5YKFN8DYDZDD in patients with GC, which is also the focus of our next research. In conclusion, the findings provided in this study of tRF-31-U5YKFN8DYDZDD could provide new insights for novel types of diagnostic biomarkers and predictors of poor prognosis. The tRF-31-U5YKFN8DYDZDD, as a representative GC-associated tsRNA, may play a tumor promoter role in GC and could serve as a potential therapeutic target.

## Data Availability Statement

The datasets presented in this study can be found in online repositories. The names of the repository/repositories and accession number(s) can be found in the article/supplementary material.

## Ethics Statement

The studies involving human participants were reviewed and approved by the ethics committee of the Affiliated Hospital of Nantong University (ethical review report number: 2018-L055). The patients/participants provided their written informed consent to participate in this study.

## Author Contributions

YH and SJ conceived the study. SJ and CP gave constructive guidance and made critical revisions. XS and CP provided the clinical knowledge and data collection of GC. YH, SQ, and MZ performed experiments. XG and HZ arranged the data and performed the statistical analysis. YH finished the manuscript and figures. All authors contributed to the article and approved the submitted version.

## Funding 

This project was supported by grants from the National Natural Science Foundation of China (No. 81600158, No. 81871720, No. 82072363) and Nantong Municipal Health Commission (QA2020027).

## Conflict of Interest

The authors declare that the research was conducted in the absence of any commercial or financial relationships that could be construed as a potential conflict of interest.

## Publisher’s Note

All claims expressed in this article are solely those of the authors and do not necessarily represent those of their affiliated organizations, or those of the publisher, the editors and the reviewers. Any product that may be evaluated in this article, or claim that may be made by its manufacturer, is not guaranteed or endorsed by the publisher.

## Abbreviations

GC, Gastric cancer; AGE, Agarose gel electrophoresis; qRT-PCR, quantitative real-time PCR; ROC, Receiver operating characteristic; CEA, Carcinoembryonic antigen; CA199, Carbohydrate antigen 199; CA724, Carbohydrate antigen 724; ncRNAs, non-coding RNA; tRNAs, transfer RNA; tsRNAs, tRNA-derived small RNAs; tRFs, tRNA-derived fragments; tiRNAs, tRNA halves; U6, RNU6B; AUC, Area under the curve; CI, Confidence interval; SD, Standard deviation; CV, Coefficient of variation; SEN, Sensitivity; SPE, Specificity; ACCU, Overall accuracy; PPV, Positive predictive value; NPV, Negative predictive value; EMT, epithelial to mesenchymal transformation; Runx 1, runt-related transcription factor 1.
